# Immune Cells Profiles In The Peripheral Blood Of Patients With Moderate To Severe COVID-19 And Healthy Subjects With and Without Vaccination With The Pfizer-BioNTech mRNA Vaccine

**DOI:** 10.3389/fimmu.2022.851765

**Published:** 2022-07-11

**Authors:** Raja’a Al-Attiyah, Hussain A. Safar, Lotfy Botras, Marina Botras, Fatma Al-Kandari, Wassim Chehadeh, Abu Salim Mustafa

**Affiliations:** ^1^ Department of Microbiology, Faculty of Medicine, Health Sciences Center, Kuwait University, Kuwait, Kuwait; ^2^ Genomics, Proteomics and Cellomics Sciences Research Unit (OMICSRU), Research Core Facility, Health Sciences Center, Kuwait University, Kuwait, Kuwait; ^3^ Mubarak Al-Kabeer Hospital, Ministry of Health, Kuwait, Kuwait

**Keywords:** COVID-19, immune response, flow cytometry, vaccination, SARS-CoV-2

## Abstract

Severe Acute Respiratory Syndrome Coronavirus 2 (SARS-CoV-2), the causative agent of Coronavirus disease 2019 (COVID-19), has caused a global crisis. Patients with COVID-19 present with a range of clinical manifestations, from no symptoms to severe illness. However, little is known about the profiles of immune cells required to protect against SARS-CoV-2. This study was performed to determine the immune cells profiles in the peripheral blood of COVID-19 patients with moderate to severe disease (n=52), and compare the findings with those from healthy subjects vaccinated with Pfizer BioNTech mRNA vaccine (VS) (n=62), and non-vaccinated healthy subjects (HS) (n=30) from Kuwait. Absolute counts and percentages of total lymphocytes and lymphocyte subsets (CD3+ T cells, CD4+ T cells, CD8+ T cells, CD19+ B cells, and CD16+CD56+ NK cells) in the peripheral blood of the three groups were analyzed using flow cytometry. The results showed that the absolute counts of total lymphocytes, CD3+, CD4+, and CD8+ T cells, CD19+ B cells, and CD56+ NK cells, were significantly lower in COVID-19 patients than normal healthy controls and vaccinated subjects. The percentages of CD3+ and CD4+ T lymphocytes were also significantly lower in the COVID-19 patients. However, the percentage of CD16+CD56+ NK cells was significantly higher in the peripheral blood of COVID-19 patients, compared to the HS and VS groups with no detectable differences in the percentages of CD8+ T cells and CD19+ B cells between the three groups. Analysis of the monocyte subsets has showed a significantly higher percentage of CD14+HLA-DR+ monocytes in COVID-19 patients compared to HS whereas the inflammatory CD14+CD16+ HLA-DR+ monocytes, and the non-classical CD16+HLA-DR+ monocytes showed significantly lower frequency in the blood of the patients than that of HS. These findings demonstrate perturbations of both innate and adaptive immune cell subsets that reflect dysregulated host responses in COVID-19 patients with moderate to severe disease.

## Introduction

In December 2019, patients in Wuhan, China presented with pneumonia of unknown etiology (1, 2). A novel coronavirus, given the name Severe Acute Respiratory Syndrome Corona Virus 2 (SARS-CoV-2), was reported as the cause of pneumonia. SARS-CoV-2, a member of the genus beta coronavirus, has spread quickly all over the world, leading to a pandemic that infected over 528,816,317 people and caused 6,294,969 deaths (as of June 3, 2022) and as of 31 May 2022, a total of 11,947,644,522 vaccine doses have been administered (3). This new pandemic has tremendously affected the global economy and put a great strain on global health care systems. The World Health Organization called this disease coronavirus disease 2019 (COVID-19). COVID-19-patients clinically present with symptoms including fever, fatigue, muscle pain, diarrhea, and pneumonia and can cause death in severe cases. The disease was found to be more severe in patients who are older and have various other co-morbidities, such as diabetes, obesity, and heart disease (4) but can also affect patients with younger age with no pre-existing medical conditions. Patients with severe disease were also shown to have abnormalities in several laboratory parameters, including elevated levels of procalcitonin, lactate dehydrogenase, increased serum levels of inflammatory markers (e.g. D-dimer, C-reactive protein), neutrophil counts, and pro-inflammatory cytokines, such as interleukin-6. Lymphopenia and thrombocytopenia are also associated with severe COVID-19 disease, viral pneumonia, multi-organ failure, and death.

What triggers a severe illness in some patients infected with SARS-CoV-2 is not completely understood, and the severe disease may not be due to the viral infection alone but it could be also attributed to a defective immune response (1, 2). An aggressive immune response to SARS-CoV-2 is thought to contribute to disease severity and death in patients with COVID-19. These patients were reported to have high levels of circulating cytokines, lymphopenia, and mononuclear cell infiltration in the lungs, heart, spleen, lymph nodes, and kidney, as detected in post-mortem studies (1, 2). There are enormous challenges that scientists face while trying to study the immunological aspects of COVID-19 due to the multiple immunological parameters that need to be measured, and the possibility of the existence of multiple pathways of protection against COVID-19 disease (5). It is also possible that correlates of protection are different at different time points after vaccination and/or with different vaccines.

Furthermore, due to the complexity of the clinical manifestations and the lack of understanding of severe COVID-19 immunopathogenesis, it has been difficult to find effective therapeutic strategies (6). There are several questions raised which include, e.g. which is more relevant to protection, cellular or humoral immunity? and how long does a protective response last? in addition to the emergence of virus variants, which is an extra challenge. A large number of studies have been carried out to analyze the immune response mounted in response to SARS-CoV-2 (4, 6–7), but some of these studies were carried out on single patients or a small number of patients at different stages of the disease or with reports on a limited number of immune cell subsets (8–11).

Thus, to address some of the above mentioned issues in the present study, a comprehensive analysis of various immune cell subsets in the peripheral blood was performed with patients having moderate to severe COVID-19 (n=52), and compared to subjects vaccinated with Pfizer BioNTech mRNA vaccine (n=62) and unexposed healthy non-vaccinated subjects (n=30) using flow cytometry. We further evaluated the activation status of T and B lymphocytes and monocyte subsets in all the groups.

## Materials and Methods

### Study Population and Sample Collection

**Figure 1:**
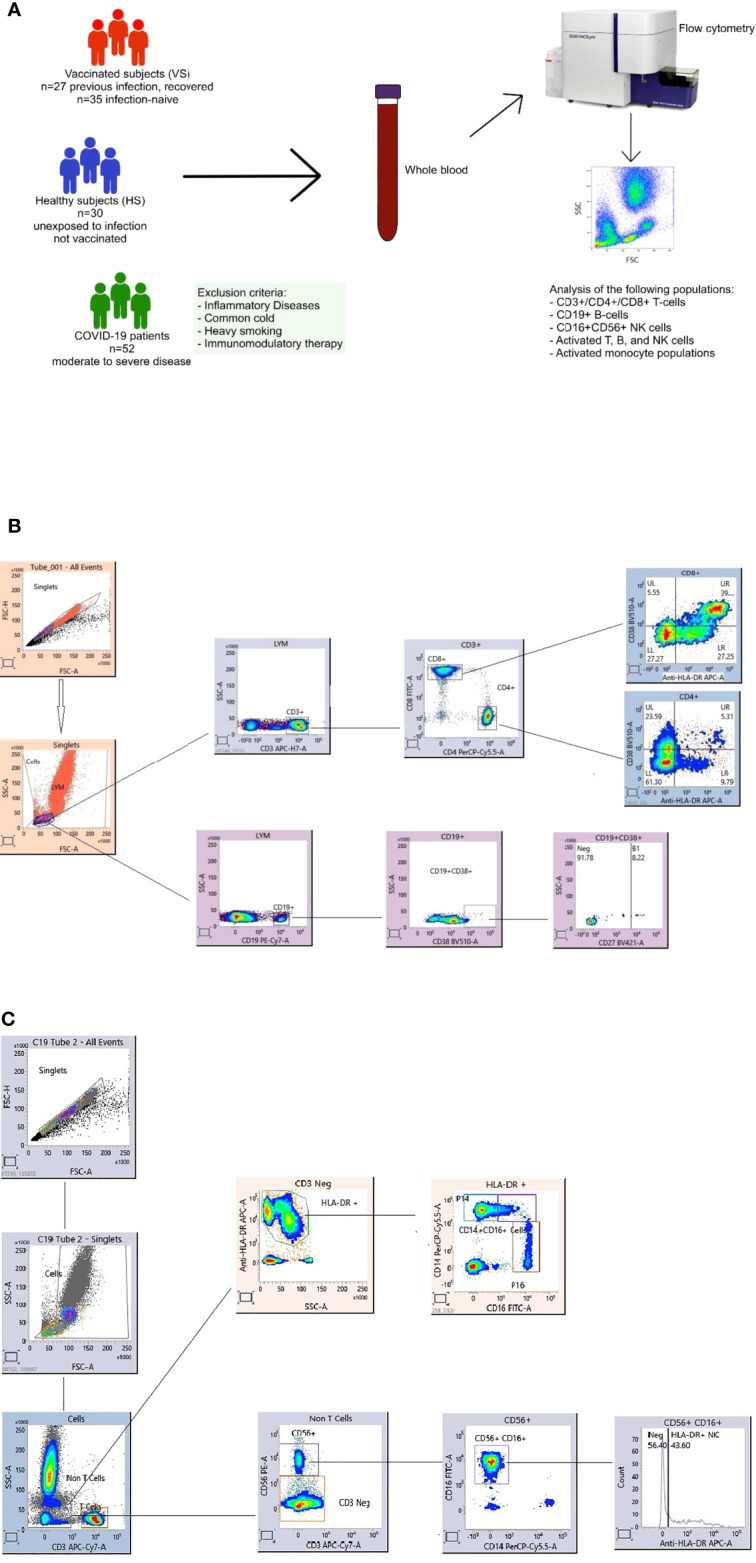
Overview of the study including healthy subjects (HS), COVID-19 patients and vaccinated subjects (VS) **(A)**. Flow cytometry gating strategy for immune cell subsets. Gating panels are shown for **(B)** CD27+CD38+CD19+ antibody-secreting B cell (ASCs, plasmablasts) and activated CD38+HLA-DR+CD4+ and CD38+HLA-DR+CD8+ T cells and **(C)** activated HLA-DR+NK cells and monocyte subpopulations. Fresh whole blood (100 microlitre per stain) was used to measure the percentage of CD3-CD19+CD27+CD38+ ASC populations, in addition to activated HLA-DR+CD38+CD8+ and HLA-DR+CD38+CD4+ T cells. Another tube of blood was stained for the percentage of inflammatory CD14+CD16+, CD16+ and conventional CD14+ monocytes and, activated HLA-DR+CD3-CD16+CD56+ NK cells. After staining the whole blood was for 20 mins at room temperature (RT) in the dark, samples were lysed with BD FACS Lysing solution, washed and fixed with 1% paraformaldehyde. All the stained blood samples were acquired on a FACS Lyric flow cytometer (BD). Flow cytometry data were analyzed using FACS Suite Research software.

The study enrolled COVID-19 patients (n=52) with moderate to severe disease admitted to Mubarak Al-Kabeer Hospital, Kuwait. All of the patients were initially diagnosed based on the clinical symptoms and later confirmed by quantitative RT-PCR (qRT-PCR) analysis of nasopharyngeal swab samples for SARS-CoV-2. Blood samples were collected from the infected patients during the months of 11.04.21 to 28.06.21. Peripheral blood was collected from infected patients before the administration of antibiotics, steroids or antiviral agents. Patients at least 15 years age were included in the study whereas patients with inflammatory diseases (e.g. myocarditis, chronic peptic ulcer, tuberculosis, rheumatoid arthritis, Crohn’s disease, active hepatitis, asthma, allergy, lupus … etc.), common cold, heavy smoking, or on medications that can inhibit the immune system, e.g. steroids, or immunosuppressive agents were excluded from the study. The infected patients included Kuwaiti as well as non-Kuwaiti citizens residing in Kuwait (**Figure 1A**).

The study also included healthy subjects (n=62) who received two doses of the Pfizer-BioNTech BNT162b2 mRNA vaccine. Blood was collected from vaccinated healthy subjects 3-14 weeks after vaccination. Age- and sex-matched non-vaccinated and COVID-19 negative healthy subjects (n=30), as confirmed by laboratory diagnosis, were also enrolled in the present study as a control group.

The following information on each patient were taken from electronic medical records: age, gender, medical history, symptoms, severity assessment on admission, laboratory findings (including CRP, lymphocyte count, D-dimer), initial laboratory investigations including a complete blood count, coagulation profile, and serum biochemical test and chest computed tomography (CT) or radiograph findings. On admission, moderate illness was defined according to the following criteria: respiratory rate ≥ 20 breaths per minute, heart rate ≥ 90 beats per minute; with saturation of oxygen (SpO2) > 93% at rest.

Severe illness was defined according to the following criteria: breathing rate ≥30 times/min, pulse oximeter oxygen saturation (Spo2) ≤93% at rest; and ratio of partial pressure of arterial oxygen (Pao2) to fraction of inspired oxygen (Fio2) ≤300 mmHg. Demographic data are summarized in **Table 1**.

**Table 1:** Demographics and Clinical Characteristics of study populations.

Parameter	Healthy Subjects	Vaccinated subjects	COVID-19 patients
*N*	30	62	52
Age (yrs)	34.8 (26-56)	34.7 (18-68)	47.8 ^a^ (15-82)
Gender, % (n)			
Male	66.7 (20)	40.3 (25)	71.2 (37) ^a^
Female	33.3 (10)	59.7 (37)	28.8 (15)
Symptoms			
Fever			23 (44.2%)
Cough			22 (42.3%)
Breath Shortness			27 (52 %)
Bilateral lung involvement			21 (40.4 %)
CBC			
Leukocytes #	(3.7-10)^b^	ND	7.4 (3.3-15.1)
Neutrophils #	(1.7-6)	ND	4.8 ((2.3-11.8)
Lymphocytes #	(1-3)	ND	1.1 (0.2-3.8)
NLR	ND	ND	5.6 (0.9-35.5)
Monocytes #	(0.2-1)	ND	0.4 (0.1-1.3)
Platelets #	(130-430)	ND	217 (105-431
Previously tested positive	0/30	27/62	Not applicable
for SARS-CoV-2			
D-dimer test, (ng/ml)	<255	ND	298 (67-3760)

CBC, complete Blood Count; NLR, Neutrophil/lymphocyte ratio, Data are median (min-max); #, 1x10^9^/L.

a statistically significant compared to healthy and vaccinated subjects (p<0.05).

b Normal range of laboratory.

c ND = Not Done.

At enrollment, 2 ml peripheral blood was collected from each study participant in tubes containing anti-coagulant EDTA and tested within three hours of withdrawal. The samples from COVID-19 patients were obtained at the time of diagnosis and before the administration of any treatment. Written informed consents from all participants were obtained before enrolling them in the study. Ethical approvals were obtained from the Ethical Committees of the Health Sciences Centre, Kuwait University, and the Ministry of Health, Kuwait.

### Antibody Staining for Immunophenotyping

The Immunophenotyping of peripheral blood for various markers of T cells, B cells, monocytes and NK cells was performed using flow cytometry according to the manufacturer’s instructions. In brief, 50 μl of whole blood was added to a Trucount tube (BD Biosciences, Franklin Lakes, NJ, USA) and analyzed for cell percentages and cell counts (cells/μL) of CD3+/CD4+/CD8+ T-cell, CD19+ B-cell, and CD16+CD56+ NK cells by multiple-color flow cytometry using 20 μl cocktail containing human monoclonal anti-CD3-FITC-A, anti-CD4-PE-CY7, anti-CD8-APC-CY7, anti-CD19-APC, anti-CD16-PE and anti-CD56-PE- and CD45 PerCP-Cy 5.5 antibodies (BD Multitest, BD Biosciences, San Jose, CA, USA). The cells were analyzed on a BD FACS Lyric flow cytometry system (BD Biosciences). A fluorescence gating strategy using CD45+ versus side scatter was carried out. Internal quality assurance was performed using optical alignment beads and BD multicheck whole blood control cells, and compensation reagents were used to eliminate bleed through fluorescence. Data analysis was performed using BD FACS Suite Clinical software (BD Biosciences).

Furthermore, analysis of the following populations (a) CD19+CD27hiCD38hi antibody-secreting B cells (ASCs, plasmablasts), (b) activated CD38+HLA-DR+ CD8+ and CD4+ T-cells, (c) activated HLA-DR+ CD3-CD16+CD56+NK cells and (d) classical CD14+ monocytes, intermediate CD14+CD16+ monocytes and non-classical CD16+ monocytes was performed by flow cytometry according to standard procedures (10). That was performed using CD45-V500-C, CD3-APC-H7, CD4-PerCP-Cy5.5, CD8-FITC, HLA-DR-APC, CD19-PE-Cy7, CD27-BV421, CD38-BV510, CD16-FITC, CD56-PE, and CD14-PerCP-Cy5.5 (BD Biosciences). In brief, whole blood was stained for 20 minutes at room temperature in the dark, then, the samples were lysed with BD FACS Lysing Solution (BD Biosciences), washed, and fixed with 1% paraformaldehyde. The samples were acquired on a BD FACS Lyric Flow Cytometry System (BD Biosciences). Physical gating was performed using CD45 staining and side scatter (SS), and the lymphocyte populations were identified to be low SS and bright for CD45 expression. Then, different lymphocyte subpopulations were identified by immunophenotyping markers. Flow cytometry data were analyzed using BD FACS Suite Research v1.3 software. Gating panels are shown in **Figure 1B** for CD27+CD38+ ASCs, activated CD38+HLA-DR+ CD8+ and CD4+ T-cells, and in **Figure 1C** for activated HLA-DR+ NK cells and CD14+, CD16+ and CD14+CD16+ monocytes.

### Correlation Plots and Heatmap Visualization

Graphpad Prism 9.2.0 (GraphPad Software, Inc., San Diego, CA, USA) was used to calculate the Pearson’s correlation coefficient to explore the correlations between age, D-dimer, the absolute count of lymphocytes, and the absolute count and the percentage of the following cell populations: CD3+, CD4+, CD8+ T cells, CD19+ B cells, CD16+CD56+ NK cells, CD4:CD8 ratio, and the percentage of CD8+CD38+HLA-DR+, and CD4+CD38+HLA-DR+ T cells, CD19+CD38+CD27+ ASCs, HLA-DR+NK cells, CD14+HLA-DR+, CD14+CD16+ HLA-DR+, and CD16+HLA-DR+ monocytes in COVID-19 positive patients and COVID-19 vaccinated subjects. The results were depicted as heat maps.

### Statistical Analysis

Statistical analyses were conducted using the Statistical Package for the Social Sciences (SPSS), release 25.0. (IBM Corp., Armonk, NY, USA). Statistical significance was set at p < 0.05. Descriptive analyses were conducted to calculate frequencies and proportions of categorical variables. Quantitative data were expressed as mean and standard error (SE). Multivariable linear regression was used to evaluate adjusted mean differences in the study outcomes across the study groups. Age, sex and gender were included in all regression models as confounders. Tukey’s Kramer test was used to correct for multiple comparisons and estimate adjusted p-values.

## Results

### Characterization of the Study Population

In the present study, immune cell profiles of hospitalized COVID-19 patients (n=52), suffering from moderate to severe disease, were evaluated. The immune cell profiles of the patients were compared to those of non-vaccinated healthy subjects (HS) (n=30) and healthy subjects vaccinated against COVID-19 using Pfizer**-**BioNTech mRNA vaccine (VS) (n=62). The median age of COVID-19 patients was 48 years (range 15-72 years), whereas that of the HS and VS were 35 (range 25-56) and 35 years (range 18-68 years), respectively. Demographic and clinical data of the study populations are presented in **Table 1**.

### Circulating Lymphocyte Subsets in the Peripheral Blood of Untreated COVID-19 Patients, Vaccinated Subjects, and Healthy Subjects

**Figure 2:**
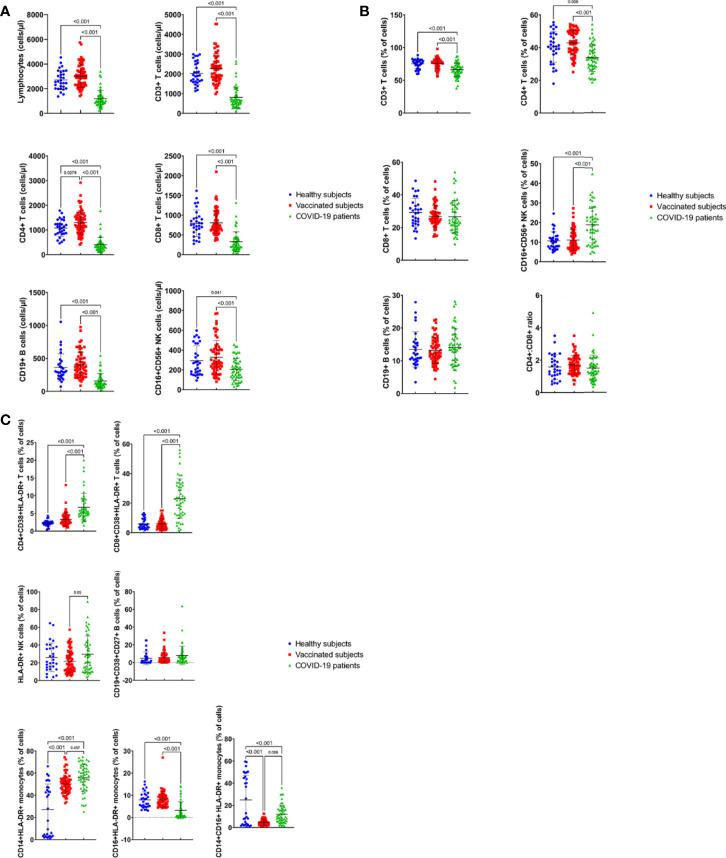
Absolut counts (cells/μl) **(A)** and percentages **(B)** of immune cell subsets in peripheral blood of COVID19-infected patients, healthy subjects and vaccinated subjects. Results are shown for total lymphocytes, CD3+, CD4+ and CD8+ T cells, CD19+ B cells and CD16+CD56+ NK cells and CD4:CD8 ratio. Each dot represents an individual donor. Data are presented as mean + SEM. **(C)** Percentage of activated immune cell subsets in peripheral blood of COVID19-infected patients, healthy subjects and vaccinated subjects. Results are shown for CD38+HLA-DR+CD4+ and CD38+HLA-DR+CD8+ T cells, CD27+CD38+CD19+ B cells and monocytes subsets: CD14+HLA-DR+, CD16+HLADR+ and CD14+CD16+HLA-DR+ cells. Data are presented as mean + SEM.

The results of the present study demonstrated that the absolute counts were significantly lower in the peripheral blood of COVID-19 patients for the following lymphocyte populations; total lymphocytes (**Figure 2A**, p < 0.001), CD3+ T lymphocytes (**Figure 2A**, p < 0.001), CD4+ T lymphocytes (**Figure 2A**, p < 0.001), CD8+ T lymphocytes (**Figure 2A**, p < 0.001), CD19+ B lymphocytes (**Figure 2A**, p < 0.001), and CD16+CD56+ natural killer cells (**Figure 2A**, p = 0.041 vs the HS and p<0.001 vs VS) compared to both the healthy subjects (HS) and vaccinated subjects (VS).

Furthermore, the percentages of mature CD3+T cells, CD4+T helper cells, CD8+T cytotoxic cells, CD19+ B cells, CD16+CD56+ NK cells were analyzed. The percentages of the following cell populations were found to be significantly lower in the infected patients, compared to the HS and VS; CD3+ T lymphocytes (**Figure 2B**, p<0.001) and CD4+ T lymphocytes (**Figure 2B**, p=0.006 vs HS and p< 0.001 vs VS). However, the percentage of CD16+CD56+ natural killer cells (**Figure 2B**, p<0.001) was found to be significantly higher in the peripheral blood of COVID-19 patients, compared to both the HS and VS (**Figure 2B**, p<0.001). Furthermore, there were no significant differences in the percentages of CD8+ T cells, and CD19+ B cells found in COVID-19 patients, compared with the HS and the VS (**Figure 2B**, p>0.05). In addition, studying CD4:CD8 ratios showed that COVID-19 patients had a similar CD4:CD8 ratio (1.5), when compared with the HS (1.6) and the VS (1.7) (**Figure 2B**, p> 0.05).

The results of the study also showed that there were no significant differences (p>0.05) between HS and VS for all the parameters tested except for the absolute count of CD4+ T cells, which was significantly higher in the VS than the HS (**Figure 2A**, p<0.05).

### Activated Lymphocyte Subsets in the Peripheral Blood of COVID-19 Patients, Vaccinated Subjects, and Healthy Subjects

The expression of CD38 and HLA-DR markers on the surface of CD4+ and CD8+ T cells reflects the activation of both cell subsets in response to viral infections. Thus, the percentages of CD4+ and CD8+ T cells expressing surface molecules, CD38 and HLA-DR, were analyzed among the study participants. The percentage of CD8+CD38+HLA-DR+ T cell population was significantly higher in the peripheral blood of COVID-19 patients (23.5%), compared to HS (6.5%) and VS (6%) groups (**Figure 2C**, p<0.001). Similarly, the frequency of CD4+CD38+HLA-DR+ T cells was significantly increased in the patients’ group (6.8%), as compared to the HS (2.3%) and VS (3.3%) (**Figure 2C**, p<0.001). Thus, the activation phenotype of both CD4+ and CD8+ T cells was confirmed by the increased co-expression of HLA-DR and CD38 in the patients compared to the HS and VS.

Furthermore, activated CD3- NK cells expressing CD16, CD56 and HLA-DR cell surface markers were detected at a significantly higher percentage in the COVID-19 patients (30.2%) compared to the VS (21.5%) (p= 0.05), but without significant difference to the HS (26%) (p> 0.05) (**Figure 2C**).

The analysis of the percentage of the CD19+CD38+CD27+ antibody-secreting B cells (ASCs) in the peripheral blood showed that there was no significant difference between COVID-19 patients (8.4%), the HS (4.5%) and the VS (5.4%) (**Figure 2C**, P>0.05).

The comparison of the HS and VS groups for the activated cell populations, CD4+ and CD8+ T cells expressing surface CD38+HLA-DR+ markers, the CD19+CD38+CD27+ B cells, and HLA-DR+CD3-CD16+CD56+ activated NK cells demonstrated the lack of significant differences between the two groups (**Figure 2C**, p>0.05).

### Monocyte Subsets in the Peripheral Blood of COVID-19 Patients, Vaccinated Subjects, and Healthy Subjects

Analysis of the changes in monocyte subsets was carried out by detecting the expression of monocyte activation markers, CD14, CD16 and HLA-DR. The detection of the classical CD14+HLA-DR+ monocytes showed a significantly higher percentage in COVID-19 patients (54.7%) compared to HS (26.7%) (**Figure 2C**, p<0.001) but not the VS (51%) (**Figure 2C**, p>0.05). This comparison has also shown that this cell population was significantly higher in the VS than the HS (**Figure 2C**, p<0.001).

However, the detection of inflammatory CD16+CD14+ HLA-DR+ monocytes, which are related to immunopathology, has shown lower frequencies of CD14+ CD16+HLA-DR+ monocytes in the blood of the patients (12.4%) than that of HS (25.1%) (**Figure 2C**, p<0.001). Furthermore, the VS had a significantly lower percentage of CD16+CD14+ monocytes (4.6%) than both of the patients (p= 0.006) and HS (p<0.001) (**Figure 2C**). In addition, the investigation of the non-classical CD16+HLA-DR+ monocytes showed that significantly lower percentage of these cells were present in COVID-19 patients (3.2%), compared with HS (8.2%) and VS (8.2%) (**Figure 2C**, P<0.001).

Furthermore, for the monocyte cell subsets, the comparison between the HS and VS has shown that there was a significant difference in the percentages of CD14+HLA-DR+ and CD14+CD16+HLA-DR+ monocytes (**Figure 2C**, p<0.001) but not the CD16+HLA-DR+ monocytes.

### The Effect of Prior Infection on Vaccine-Induced Immune Responses

Twenty-seven out of 62 VS evaluated in this study were diagnosed as COVID-19 positive with a PCR test and recovered from a mild disease (recovered) 3-6 months before receiving the two doses of the vaccine. The immune cell profiles of these subjects were compared to vaccinated subjects who were never exposed to the infection (naïve, n = 35). This comparison showed that the two groups had similar results for the absolute counts and percentages of all immune cell subsets tested in the study (**Figures 1 Supplemental A** **,** **B**). However, the percentage of CD8+ T cells was significantly lower in recovered subjects than the naïve subjects whereas the CD4:CD8 ratio was significantly higher in the recovered subjects (1.9) than in the naïve subjects (1.6) (**Figure 1 Supplemental B**, p<0.05).

Furthermore, analysis of the percentage of activated immune cells in the peripheral blood i.e. T and B lymphocytes, NK cells and monocyte subsets showed insignificant differences between the recovered and naïve subjects (**Figure 1 Supplemental C**).

**Figure 3:**
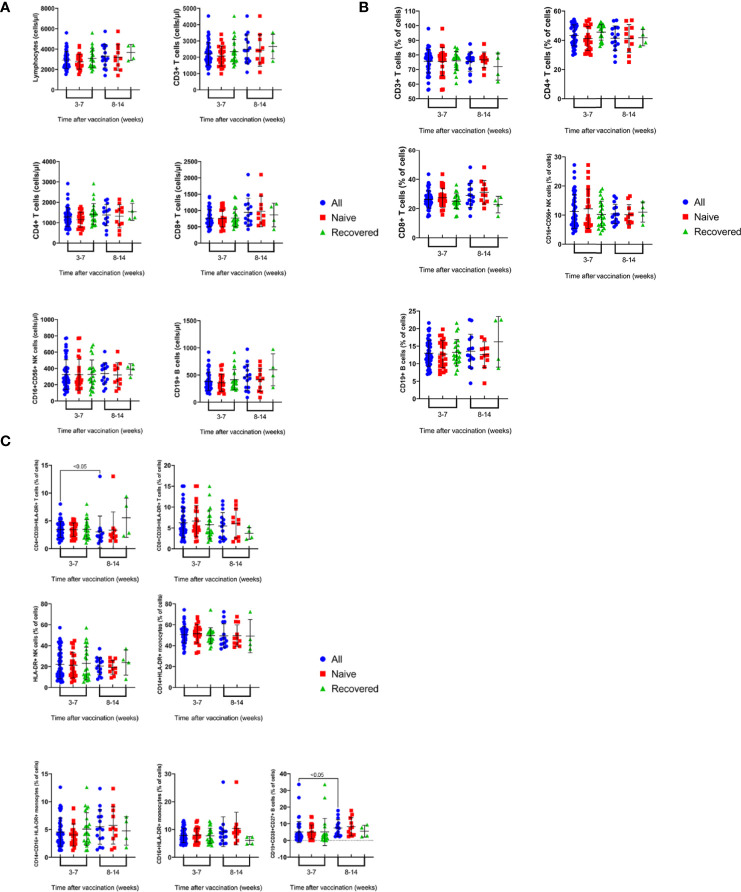
Time course of changes in the absolute counts **(A)** and percentages **(B)** of immune cell subsets in the peripheral blood of recovered-vaccinated vs naïve vaccinated subjects over 3-14 weeks post vaccination. Results are shown for total lymphocytes, CD3+, CD4+ and CD8+ T cells, CD19+ B cells and CD16+CD56+ NK cells and CD4:CD8 ratio. Each dot represents an individual donor. Data are presented as mean + SEM. **(C)** Time course of changes in the percentages activated immune cell subsets in peripheral blood of recovered-vaccinated vs naïve vaccinated subjects over 3-14 weeks post vaccination. Results are shown for CD38+HLA-DR+CD4+ and CD38+HLA-DR+CD8+ T cells, CD27+CD38+CD19+ B cells and monocytes subsets: CD14+HLA-DR+, CD16+HLADR+ and CD14+CD16+HLA-DR+ cells. Data are presented as mean + SEM.

The present study also investigated the effect of post-vaccination period among the vaccinated subjects, with and without prior infection, on the absolute counts and percentages of various immune cell subsets. Blood samples were collected from the vaccinated subjects after 3-7 and 8-14 weeks post- vaccination after receiving the second dose of the vaccine. It was demonstrated that vaccination of recovered and naïve subjects resulted in similar values of the absolute counts (**Figure 3A**) and percentages (**Figure 3B**) of immune cell subsets tested after 3-7 and 8-14 weeks of vaccination. However, the total number of vaccinated subjects showed significantly lower percentage of CD4+CD38+HLA-DR+ T cells after 8-14 weeks (**Figure 3C**, 3%) than 3-7 weeks of vaccination (**Figure 3C**, 4%) (p<0.05). On the contrary, after 8-14 weeks of vaccination, the VS showed a significantly higher percentage of CD19+CD27+CD38+ B cells (7%) than after 3-7 weeks (6%) (**Figure 3C**, p<0.05).

### Correlation Studies

**Figure 4:**
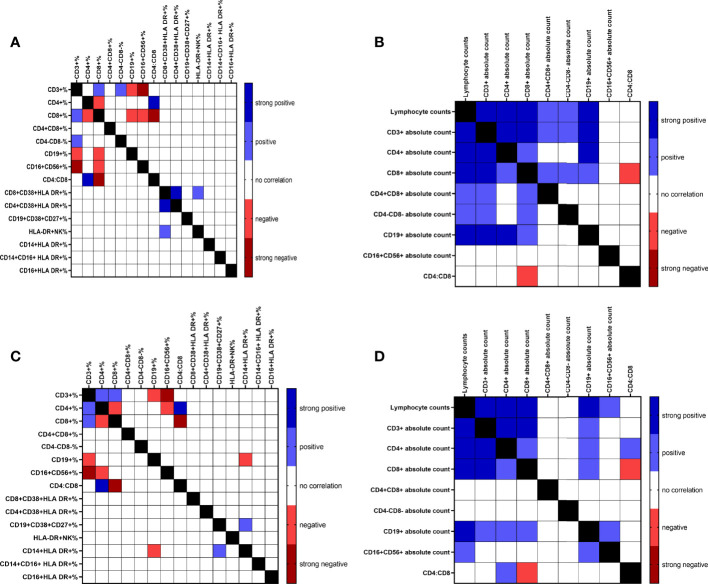
Correlation heat map of all measured immune cell populations percentage and absolute counts of COVID-19 infected patients (**A** & **B**, respectively) and percentage and absolute counts of vaccinated subjects (**C** & **D**, respectively).

Possible associations between the percentages and absolute counts of immune cell subsets were investigated in COVID-19 patients (**Figures 4A, B**) and the vaccinated subjects (**Figures 4C, D**). This demonstrated positive and negative correlation between various parameters. The Pearson’s correlation coefficient for positive (blue; r≥0.4-0.69 and p<0.05-p<0.00001), strong positive (navy; r≥0.7 and p<0.00001), negative (pink; r≥0.4-0.69 and p<0.05-p<0.00001), and strong negative (red; r≥0.7 and p<0.00001) are shown in the heat-maps (**Figures 4A–D**). We found strong positive correlation between lymphocytes counts and CD3+, CD4+, CD8+ T cells, and CD19+ B cells absolute counts, and strong negative correlation between the percentages of CD16+CD56+ NK cells and CD3+ T cells in both COVID-19 patients and the vaccinated individuals. Interestingly, in COVID-19 patients, the percentage of CD8+CD38+HLA-DR+ T cells had strong positive correlation with that of CD4+CD38+HLA-DR+ T cells (r=0.726, p<0.0001) which was not detected in the vaccinated individuals. Besides, a positive correlation between the percentages of CD8+CD38+HLA-DR+ T cells and HLA-DR+NK cells (r=0.529, p<0.0001) was demonstrated in COVID-19 patients but not in the vaccinated individuals.

## Discussion

COVID-19 is a respiratory infectious disease that is caused by severe acute respiratory syndrome coronavirus 2 (SARS-CoV-2). Respiratory droplets are the primary mode of transmission for SARS-CoV-2 (12). Understanding the roles of different immune cell subsets in protection or pathogenesis is crucial for the prevention and treatment of COVID-19. The goal of the present study was to compare the immune cell profiles in COVID-19 patients with moderate to severe disease, to that in subjects vaccinated with Pfizer BioNTech BNT162b2 mRNA COVID-19 vaccine (VS), who were unexposed to the infection, (naïve subjects) and those who recovered from COVID-19 infection followed by vaccination (recovered), all compared to unexposed healthy subjects (HS). Peripheral blood was collected from COVID-19- infected patients at enrollment (approximately 24 to 72 hours after admission), HS, and VS 3-7 and 8-14 weeks post vaccination. The evaluation of the immune cells profile was carried out by multiparametric flow cytometry to study both innate and adaptive immune cell populations. Detection of the following cell types was carried out; CD3+, CD4+ and CD8+ T cells, CD19+ B cells and CD16+CD56+ natural killer cells. In addition, the expression of activation markers, CD38 and HLA-DR on the surface of CD4+ and CD8+ T cells, CD38 and CD27 on the surface of B cells, HLA-DR molecules on NK cells, and the activated monocyte subsets were analyzed. The results demonstrated a dysregulated immune cells profile in COVID-19-patients compared to both healthy subjects (HS) and those vaccinated with Pfizer BioNTech mRNA vaccine (VS). This dysregulation was demonstrated to be statistically significant due to the reduction in both absolute counts and percentages of total lymphocytes, and the lymphocyte subsets including CD3+, CD4+ and CD8+ T cells, CD19+B cells, and CD16+CD56+ natural killer cells in the patients compared to both HS and VS (**Figures 2A, B**). However, the percentages of CD8+ T cells and CD19+ B cells were insignificantly different in the patients compared to the healthy subjects (HS) and vaccinated subjects (VS). A recent study by Sushentseva et al, 2022 has shown that the COVID-19 patients show a better survival when they have a good pool of specific CD8+ T cells (13).

Similar findings of low count of WBCs and various lymphocyte subpopulations were reported in several recent studies (7, 14–17). The detection of immune cells in the peripheral blood at low counts in response to viral infections is attributed to the migration of immune cells to the site of infection or due to the virus-mediated destruction of T cells (2). In addition, few studies of SARS-CoV-2 infection found that T cells specifically correlate with protection and decreased total lymphocytes, T lymphocyte, and B lymphocyte were associated with severe illness (7, 18, 19) and this decrease is probably caused by a defect in the induction of acquired immunity (20). In a recent study, the frequency of SARS-CoV-2 specific T cells was equal in vaccinated subjects and patients recovered from COVID-19 (13).

CD4:CD8 ratio in normal individuals is 2:1 and it gets inverted to 1:1 in some viral infections due to the increase in CD8+ T cells as shown by research into the immune response (15). Thus, this ratio can act as a diagnostic marker of the disease (15). Therefore, in the present study, possible alterations in CD4:CD8 was investigated. The present study demonstrated the absence of a significant difference in CD4:CD8 ratio between the infected patients and both the healthy and the vaccinated groups (p>0.05, **Figure 2B**). This is supported by the results of several recent studies carried out showing that in COVID-19 patients the CD4:CD8 ratio is in the normal range in spite of low CD4+ and CD8+ T cells (7, 15, 17, 21).

Examination of the immune cell profile of VS has shown that both absolute counts and percentages were not statistically different from that of the HS (**Figures 2A, B**) for all immune cell subsets tested; except for the absolute count of CD4+ T cells which was significantly higher in the VS compared to the infected patients (p< 0.001) and the HS (**Figure 2A**, p<0.05). However, the responses of VS (absolute counts and percentages) are all statistically higher than the responses of infected patients except for the percentage of CD8+ T cells and CD19+ B cells and the CD4:CD8 ratio, no difference was detected. This is supported by the finding that Pfizer BioNTech mRNA vaccine induces a strong CD8+ T cell response to various viral epitopes, a response similar to infection (22).

Natural killer (NK) cells are innate lymphoid cells that play a role in the cytolytic killing of virus-infected cells. It is not known yet if NK cells play a direct antiviral role against coronaviruses. As stated previously, the absolute count of CD16+CD56+ NK cells was found in the present study to be statistically lower in the peripheral blood of infected patients compared to both HS (p<0.05) and VS (p<0.001) (**Figure 2A**) whereas the percentage of CD16+CD56+ NK cells was statistically higher in the infected patients compared to both the HS (p<0.001) and VS (p<0.001) (**Figure 2B**). In a recent study, CD3-CD56+ natural killer cells were found to be significantly lower in the severe group compared to the healthy subjects whereas the asymptomatic group had higher levels (7, 18, 20, 23). In addition, Zingaropoli et al. (11) demonstrated that lower percentage of peripheral blood NK cell was found in COVID-19 patients compared to HS, although not statistically significant. Thus, variable findings regarding the numbers of NK cells in COVID-19 patients were demonstrated recently. The lower percentage of CD56+ NK cells found in COVID-19 patients is attributed to their migration to tissues and secondary lymphoid organs where they fight against invading pathogens (11). The high percentage of NK cells in the peripheral blood of COVID 19- patients in this study was associated with significantly higher percentage of activated CD3-CD16+CD56+ NK cells expressing HLA-DR molecules compared to the VS (p< 0.05) but with no significant difference to the HS (**Figure 2C**). This is consistent with the finding that NK cells are activated in the peripheral blood of COVID 19 patients, assessed by analyzing the expression of Ki-67, CD57, HLA-DR and CD69 surface molecules (9, 11). NK cells are important players in the innate immune response and play an essential role in fighting viral infections by directly destroying infected cells but can also contribute to immunopathology (9).

The present study shows that the absolute count of CD19+ B cells was significantly reduced in the infected patients compared to the HS (p<0.001, **Figure 2A**) and VS (p<0.001, **Figure 2A**). This is consistent with results of a previous recent study by Mathew et al. (24). However, the percentage of CD19+ B cells (**Figure 2B**) was similar in the COVID 19 patients, HS and VS groups tested (**Figure 2C**). This is consistent with the findings of Kuri-Cervantes et al (6), who showed that there is only a marginal difference in total B cells between the COVID-19 patients and the healthy subjects. On the other hand, other recent studies showed that the percentage of CD19+CD38+ B cells is lower in patients with severe COVID-19 infection (7, 19) and even when detected, it is found to be absent in 20% of the patients compared to controls (24). Antibody secreting cells, CD27+ CD38+ CD19+ B cells (ASCs, plasmablasts), is another immune cell subset that was evaluated in the present study. ASCs have been previously demonstrated to be responsible for the rapid production of antibodies following an infection with Ebola virus and infection with/and vaccination against influenza virus (10). However, it is still unknown if these active B cells are sufficient or functional or can synthesize the neutralizing antibodies required for fighting the SARS-CoV-2. CD19+CD38+CD27+ antibody secreting cells were shown, in the present study, to be similar in the COVID 19 patients, HS and VS groups tested (**Figure 2C**). Hyper activation of B cells and an increase in peripheral plasma cells expressing high CD27+ CD38+ was shown to be a sign of poor prognosis (20). In another previous study, ASCs were detected at a higher level in the blood of a single patient with mild to moderate disease at the time of viral clearance, than in healthy controls (10). In a study carried out by Kuri-Cervantes et al. (6), only marginal differences were detected in the proportions of total B cells between the COVID-19 patients and the HS, but B cell plasmablasts were significantly expanded in severe COVID-19 patients compared to HS, to the extent exceeding what was observed in other viral infections e.g. dengue and Ebola infections. The observation in the present study that the level of ASCs in the blood of COVID-19- patients was not increased could be attributed to the state of the disease in our patients or that the some of the previous studies which showed an increase in the percentage of the ASCs investigated a small number of patients. Furthermore, the level of CD27^+^CD38^+^ ASCs was found to be increased during acute viral infections or vaccination, but found to be only transiently detectable in the blood (25, 26). Most patients demonstrate seroconversion 7 to 14 days following infection, during which increased plasmablasts are detected (6). Antigen-specific B cell responses in the peripheral blood of individuals who received two doses of BNT162b2, a mRNA-based vaccine encoding full-length SARS-CoV-2 spike (S) gene, have shown that circulating IgG- and IgA-secreting ASCs specific to the S protein peaked one week after the second immunization and then declined, becoming undetectable three weeks later (27).

Most acute viral infections have been shown to induce proliferation and activation of CD8 T cells reflected in the co-expression of CD38 and HLA-DR (24). Furthermore, the association of CD38 molecule with other cell surface markers such as CD4, CD19 and class II MHC is associated with cell signaling (27). Thus, it was decided to evaluate the expression of CD38 and HLA-DR molecules on the surface of CD4 and CD8 T cells. Such analysis carried out in this study, has shown that the percentage of both CD38+ HLA-DR+ CD4+ and CD8+ T cell subpopulations is significantly higher in the peripheral blood of COVID-19 patients, compared to HS and VS control groups (**Figure 2C**). This T cell activation was very heterogeneous among the severe COVID-19 patients, reaching to baseline in some patients. SARS-CoV-2 infection was found to be associated with CD8 T cell activation in a subset of patients by Mathew et al. (24). The absence of these activated cells in some patients could be attributed to lymphopenia observed in the severe COVID-19 patients, and the possibility that activated T cells are migrating to the lung in response to the virus (6). Furthermore, CD38+HLA-DR+ CD4+ T cells were detected at high frequency in COVID-19 respiratory samples than in blood samples (28). This high frequency of activated respiratory T cells was shown to be associated with a better survival rate in COVID-19 (28). An increase in the percentage of CD38+ and HLA-DR+ memory CD4+ and CD8+ T cells was also detected in severe COVID-19 patients compared to healthy subjects in other recent studies and this correlated with poor prognosis (6, 20, 24, 29). Consistent with the findings of this study, the activation of those cell subsets was shown to be also highly heterogeneous in the COVID-19 patients (6). In addition, as shown in the correlation studies carried out for our patients (**Figure 4A**), the activation of CD4 T cells was found to correlate with the activation of CD8 T cells which agrees with the findings by Mathew et al. (24). A significant increase in HLA-DR^+^CD38^+^ non-naïve CD8 T cells has also been reported in hospitalized COVID-19 patients compared to HS and recovered donors (24–26), although many patients showed little evidence of T cell activation in the blood. Furthermore, in a patient with mild to moderate disease, the frequency of CD38+HLA-DR+ CD8+ and CD38+HLA-DR+ CD4+ T cells was much higher than in healthy individuals (10). However, another recent study has shown that the percentage of activated HLA-DR+CD3+ T cells was lower in patients with severe COVID-19 compared to healthy subjects (7).

Monocytes are important cells that participate in the production of immune responses against pathogens. There are three distinct blood monocyte subsets defined by the expression of CD14 and CD16; immature classical (CD14+CD16-), more differentiated inflammatory transitional or intermediate (CD14+CD16+), and non-classical (CD14dimCD16+) (7, 30). Classical monocytes play a role in phagocytosis, immune responses, and migration whereas intermediate monocytes are responsible for antigen presentation, and non-classical monocytes are responsible for the antiviral responses. High percentage of both CD14+CD16+ and the CD16+ monocytes were detected during inflammation (7). In addition, monocytes/macrophages play an important role in the production of both innate and adaptive immune responses (7). It has been also reported earlier that monocytes are the main players of the cytokine storm in COVID-19 infection. Low level of monocytes and T lymphocytes was detected in the peripheral blood of COVID-19 patients and was shown to be due to their immigration into the infected site which results in the immunopathogenesis of COVID-19 (7).

This study analyzed the different subsets of HLA-DR+ activated monocytes in the peripheral blood of infected patients compared to both the HS and VS. The HLA-DR+CD14+ classical monocytes were significantly higher in infected patients (p<0.001) and VS (p<0.001) compared to HS (**Figure 2C**). However, the level of non-classical HLA-DR+CD16+ monocytes were at significantly lower level in the infected patients when compared to both the VS and HS (p<0.001 and p<0.001, respectively, **Figure 2C**). Furthermore, the inflammatory CD14+CD16+ monocytes expressing HLA-DR molecules were at significantly higher level in HS than its level in both of the patients and VS (p<0.001 and p<0.001, respectively, **Figure 2C**). The infected patients also produced higher level of CD14+CD16+ cell subset than VS (p= 0.006, **Figure 2C**). In normal physiological conditions, 85% of the monocytes in the peripheral blood express CD14++ CD16 low HLA-DR++ phenotype and these cells leave the circulation and infiltrate to the inflammatory site following infection (20). Various studies have been carried out recently to study the level of monocyte subsets in response to COVID-19 infection. A higher percentage of CD14+CD16+ inflammatory monocytes was demonstrated in patients with mild to severe COVID-19 (20, 31). They also showed that there is a significant expansion of CD14+CD16+monocytes producing IL-6 in the peripheral blood of ICU COVID-19 patients than those who did not require ICU hospitalization. Furthermore, CD14+CD16+ monocytes were detected at high frequency in COVID-19 respiratory samples than in blood samples (28). However, non-classical monocytes expressing CD16 at high intensity were lower in patients with severe COVID-19 rather than the inflammatory monocytes (20, 31). In another recent study by Gatti el al. (8), a significant decrease in non-classical CD16+ monocytes and CD14+CD16+ intermediate monocytes was detected in patients with severe SARS-CoV-2 infection. Increased level of both cell subsets were reported in patients with moderate disease. Furthermore, a study of a Spanish cohort led by Sánchez-Cerrillo (30) demonstrated that all circulating myeloid subsets were significantly reduced in the peripheral blood of COVID-19 patients. This is associated with the migration of CD14+CD16+ inflammatory transitional and CD14 dim CD16+ non-classical monocytes from the blood to lungs in patients with severe COVID-19 without showing a significant correlation between total CD16+ monocytes, both non-classical and inflammatory cells, and disease severity. Kuri-Cervantes et al (6), reported that the total percentage of CD14+ HLA-DR+ monocytes, as well as monocyte subsets, were similar across groups of COVID-19 patients and healthy subjects whereas kudryaavtsev et al. (20) demonstrated high number of CD14++ HLA-DR++ activated monocytes in patients with mild disease. In addition, HLA-DR+ CD16+ monocytes existed at large numbers in the lungs of critical COVID-19 patients and so they could contribute to the disease (30). Variation detected in the level of monocyte subsets in different studies could be due to differences in the disease severity of the patients tested. In addition, the present study reported findings of activated monocyte subsets that express HLA-DR molecule on its surface.

In the present study, 62 vaccinated subjects were included out of which 27 subjects were previously infected with SARS-CoV-2, recovered and then vaccinated 4-6 months following recovery. A comparison was carried out to evaluate the immune response of the recovered vaccinated and the naïve vaccinated subjects who were never exposed to the infection (n=35). Results have shown that the two groups produced similar levels of absolute counts and percentages of total lymphocytes, and various other immune cell types (**Figures 1 Supplemental A** **–** **C**). This is except for the percentage of CD8+ T cells (p<0.05, **Figure 1 Supplemental B**) which was higher in the naïve group (p<0.05, **Figure 1 Supplemental C**) whereas the CD4:CD8 ratio was higher in the recovered group (p<0.05, **Figure 1 Supplemental B**). This is consistent with the results of a recent study which demonstrated that both recovered and naïve groups produced similar humoral and cellular responses (32). In another recent study by Kuri-Cervantes et al. (6), the percentage of T cells and NK cells were found to be similar in the recovered group and the healthy subjects. This could lead to the conclusion that natural infection followed by vaccination or vaccination in naïve subjects can act similarly as an immune stimulus. However, one need to compare the level of the antigen-specific cells in both vaccinated groups.

The present study also carried out a comparison of the immune cell profile of the recovered and naïve subjects at 3-7 and 8-14 weeks following vaccination. The comparison demonstrated that there are no significant differences between the two groups in absolute counts and percentage of CD3+, CD4+ and CD8+ T cells, CD4:CD8 ratio, CD19+ B cells and NK cells (**Figures 3A, B**). Furthermore, no differences were noted between the two groups when the percentages of activated CD38+HLA-DR+CD8+T cells and HLA-DR+NK cells were analyzed (**Figure 3C**), whereas the percentage of CD38+HLA-DR+CD4+ T cells was significantly higher at 3-7 weeks compared to its level at 8-14 weeks of vaccination (p<0.05, **Figure 3C**). Furthermore, CD38+CD27+CD19+ ASCs were at a higher percentage 8-14 weeks than 3-7 weeks of vaccination (p<0.05, **Figure 3C**). A recent study demonstrated that circulating plasma cells secreting antibodies specific to S protein of SARS-CoV-2 peaked one week after the second immunization and then declined, becoming undetectable three weeks later (27).

In this study, there were no differences in most of the mounted immune responses between the naïve and recovered vaccinated subjects at 3-7 and 8-14 weeks post vaccination (**Figures 3A–C**). A recent study by Lozano-Ojalvo et al (32), has shown that the administration of two doses of Pfizer BioNTech mRNA vaccine leads to the production of similar humoral and cellular responses in both recovered and naïve groups. This similarity in the type of immune response produced following vaccination or infection followed by vaccination indicates that mRNA vaccines are able to induce immune response equivalent to that induced by infection. More detailed comparative studies are required, but a preliminary work has indicated that Pfizer-BioNTech BNT162b2 mRNA vaccine strongly induced SARS-CoV-2 specific CD8+ T cells, which was equivalent to natural infection (24). It has also been shown that there is no major difference in frequency and phenotype of memory T cells generated by natural infection and vaccination (22). In addition, infection followed by vaccination has been shown to induce an expansion of the existing spike-specific responses (22) and can lead to a more durable response (33). The pre-existing T cells in recovered patients was found to correlate with a better cellular and humoral response following mRNA vaccination (33). Thus, it would be interesting to study the duration of the protective response induced following vaccination in the recovered vs the infection-naïve subjects (33) as BNT162b2 is the first mRNA vaccine used on a large scale (34).

Interestingly, in COVID-19 patients, the percentage of CD8+CD38+HLA DR+ T cells demonstrated strong positive correlation with the percentage of CD4+CD38+HLA DR+ T cells (r=0.726, p<0.0001), which was not detected in the vaccinated subjects. A recent study shows that activation of CD4+ and CD8+ T cells expressing CD38+ and HLA-DR+ surface molecules was associated with each other and with the percentage of plasmablasts in patients with moderate and severe COVID-19 disease (6).

Elimination of a viral infection requires the interaction of various immune cell types including CD4+ T cells, CD8+ T cells, B cells NK cells and monocytes. Several previous studies and the present study demonstrate that the decreased immune cells subsets in SARS-CoV-2 infection is related to disease severity (7, 14, 19, 35, 36). Some recent studies suggested that the mechanism of lymphopenia is T cell exhaustion or dysfunction (24, 36). Other studies suggest that a strong immune response is produced which leads to immunopathology (16, 24). Autopsies showed high numbers of the virus in the respiratory tract and other tissues, which suggests ineffective immune responses (24, 37).

The dissection of the specific immune response is essential to find out correlates of protection against SARS-CoV-2 (24). This will also help in the identification of proper vaccination protocols that can help preventing pandemic recurrence due to new SARS-CoV-2 variants. Future studies can also investigate the use of lymphocyte subset counts or other immunological perturbations as prognostic markers of disease severity, mortality, and response to treatment in patients infected with SARS-CoV-2.

There are several limitations in the present study which include the predominance of males in the patients and healthy subject groups whereas females predominated the vaccinated group. In addition, both the HS and VS groups were younger than individuals with COVID-19 disease. We were also limited in the number of healthy individuals due to the difficulty getting healthy subjects who are not vaccinated at the time of peak pressure of SARS-CoV-2 infection. Another important limitation of the present study is that we could not compare the effect of one vs two doses of Pfizer BioNTech mRNA vaccine in individuals with pre-existing immunity. An infection with COVID-19 was shown to enhance both the cellular and humoral immune response to vaccination (38). Thus, it has been suggested in several recent studies that the second vaccine dose in recovered subjects with pre-existing immunity does not further increase their humoral immune response from the first dose, although the effect on T cells has not been studied yet (32, 39–42). More follow-up studies of the antigen-specific immune response in vaccine-only, infection-only, and vaccinated after infection groups should be carried out, in addition to the performance of functional studies of both T and B cells. Lastly, the cytokine storm syndrome was not evaluated in the current study.

## Data Availability Statement

The original contributions presented in the study are included in the article/**Supplementary Material**. Further inquiries can be directed to the corresponding author.

## Ethics Statement

Ethical approvals were obtained from the Ethical Committees of the Health Sciences Centre, Kuwait University, and the Ministry of Health, Kuwait. The patients/participants provided their written informed consent to participate in this study.

## Author Contributions

RA-A, HAS, and AM contributed to the conception and design of the study. RA-A, HAS, and AM acquired the Funding. LB, MB, and FA provided the clinical material. RA-A and HAS were responsible for the investigations. HAS performed the statistical analysis. RA-A and HAS finalized the figures and tables. RA-A and HAS wrote the first manuscript draft. RA-A, HAS, WC, and AM reviewed and edited the final manuscript draft. All authors contributed to the article and approved the submitted version.

## Funding

This work was supported by Kuwait Foundation for the Advancement of Sciences (KFAS), project No PN-20-13MI-06.

## Conflict of Interest

The authors declare that the research was conducted in the absence of any commercial or financial relationships that could be construed as a potential conflict of interest.

## Publisher’s Note

All claims expressed in this article are solely those of the authors and do not necessarily represent those of their affiliated organizations, or those of the publisher, the editors and the reviewers. Any product that may be evaluated in this article, or claim that may be made by its manufacturer, is not guaranteed or endorsed by the publisher.

## Supplementary Material

Figure 1 Supplemental Supplemental Absolut counts (cells/microliter) **(A)** and percentages **(B)** of immune cell subsets in peripheral blood of recovered-vaccinated vs naïve vaccinated subjects. Results are shown for total lymphocytes, CD3+, CD4+ and CD8+ T cells, CD19+ B cells and CD16+CD56+ NK cells and CD4:CD8 ratio. Each dot represents an individual donor. Data are presented as mean + SEM. **(C)** Percentage of activated immune cell subsets in peripheral blood of recovered-vaccinated vs naïve vaccinated subjects. Results are shown for CD38+HLA-DR+CD4+ and CD38+HLA-DR+CD8+ T cells, CD27+CD38+CD19+ B cells and monocytes subsets: CD14+HLA-DR+, CD16+HLADR+ and CD14+CD16+HLA-DR+ cells. Data are presented as mean + SEM.
