# Supplementary figures and images for: Immune Cells Profiles In The Peripheral Blood Of Patients With Moderate To Severe COVID-19 And Healthy Subjects With and Without Vaccination With The Pfizer-BioNTech mRNA Vaccine
Source: Front Immunol. 2022 Jul 11;13:851765. doi: 10.3389/fimmu.2022.851765 (PMC9309529; doi:10.3389/fimmu.2022.851765)

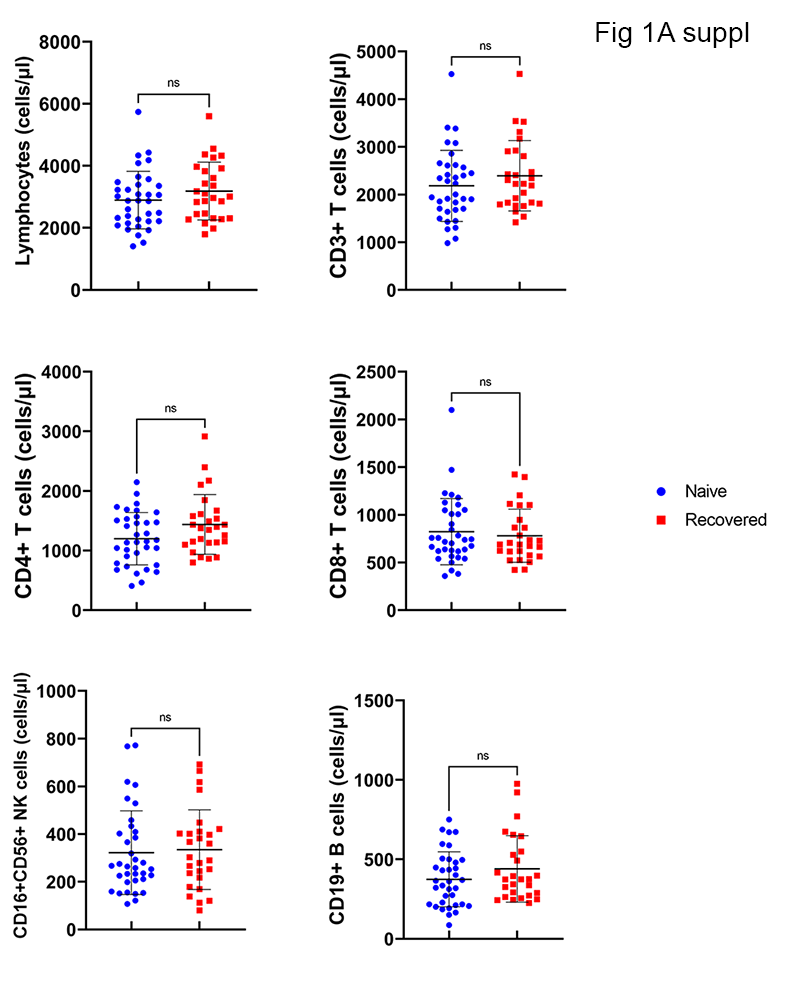

Supplement: Figure 1 Supplemental — Supplemental Absolut counts (cells/microliter) (A) and percentages (B) of immune cell subsets in peripheral blood of recovered-vaccinated vs naïve vaccinated subjects. Results are shown for total lymphocytes, CD3+, CD4+ and CD8+ T cells, CD19+ B cells and CD16+CD56+ NK cells and CD4:CD8 ratio. Each dot represents an individual donor. Data are presented as mean + SEM. (C) Percentage of activated immune cell subsets in peripheral blood of recovered-vaccinated vs naïve vaccinated subjects. Results are shown for CD38+HLA-DR+CD4+ and CD38+HLA-DR+CD8+ T cells, CD27+CD38+CD19+ B cells and monocytes subsets: CD14+HLA-DR+, CD16+HLADR+ and CD14+CD16+HLA-DR+ cells. Data are presented as mean + SEM. [file Image_1.tif]

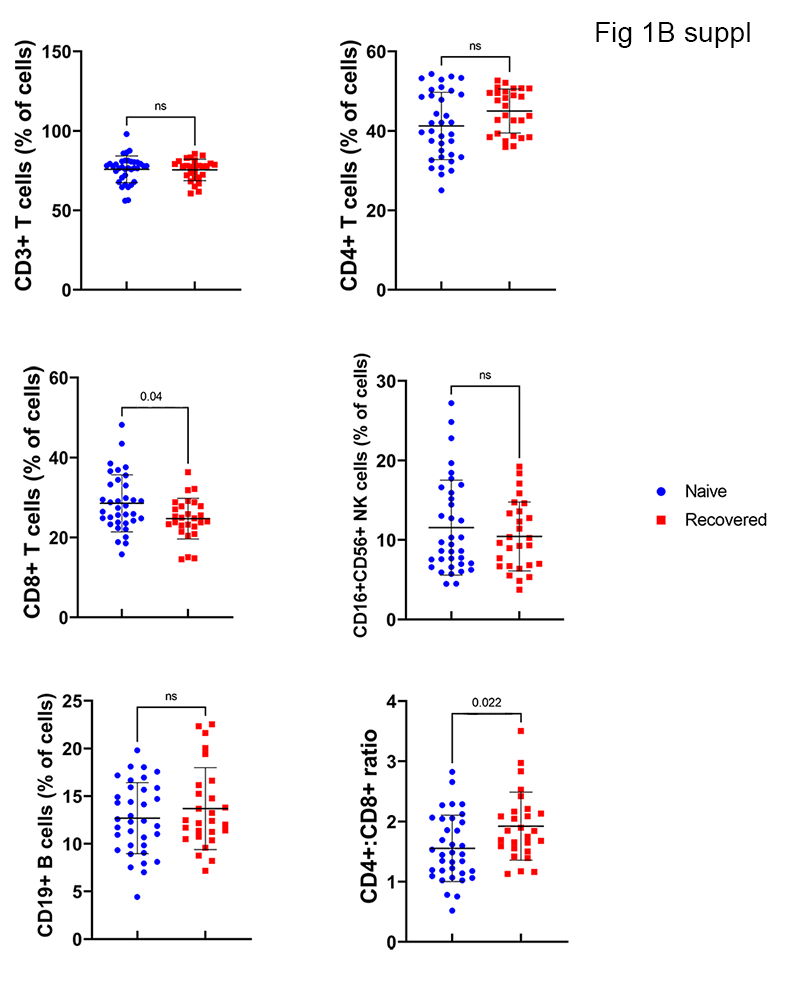

Supplement: Supplementary file 2 [file Image_2.tif]

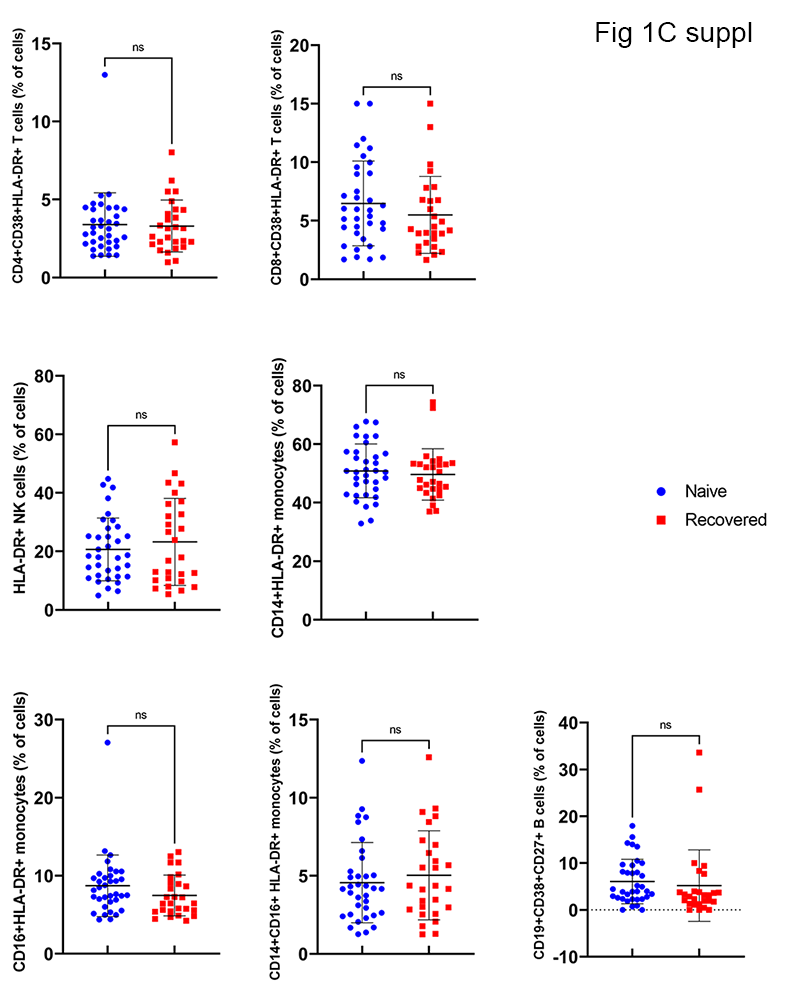

Supplement: Supplementary file 3 [file Image_3.tif]
